# Sivelestat Attenuates Myocardial Reperfusion Injury during Brief Low Flow Postischemic Infusion

**DOI:** 10.1155/2013/279847

**Published:** 2013-05-22

**Authors:** Sverre E. Aune, Steve T. Yeh, Periannan Kuppusamy, M. Lakshmi Kuppusamy, Mahmood Khan, Mark G. Angelos

**Affiliations:** ^1^Department of Emergency Medicine, The Davis Heart Lung Research Institute, The Ohio State University Wexner Medical Center, 750 Prior Hall, 376 W. 10th Avenue, Columbus, OH 43210, USA; ^2^The Division of Cardiovascular Medicine, The Davis Heart Lung Research Institute, The Ohio State University Wexner Medical Center, Columbus, OH 43210, USA

## Abstract

The neutrophil elastase inhibitor sivelestat (ONO-5046) possesses unknown mechanisms of cardioprotection when infused following global ischemia, even in the absence of neutrophils. Since myocardial ischemia-reperfusion injury is strongly associated with endothelial dysfunction and reactive oxygen species (ROS) generation during reperfusion, we have tested the hypothesis that infusion of sivelestat during postischemic low flow would preserve endothelial and contractile function and reduce infarct size through an ROS-mediated mechanism. Isolated male rat hearts, subjected to global ischemia of 25 minutes, were reperfused with low flow with or without sivelestat followed by a full flow reperfusion. Hearts treated with sivelestat showed a significant improvement of LV contractile function and a reduction in infarct size. Infusion of L-NAME (nonspecific blocker of endothelial nitric oxide synthase (eNOS)) along with sivelestat during reperfusion reversed the preservation of contractile function and infarct size. *In vitro *EPR spin trapping experiments showed that sivelestat treatment decreased superoxide adduct formation in bovine aortic endothelial cells (BAECs) subjected to hypoxia-reoxygenation. Similarly, dihydroethidine (DHE) staining showed decreased superoxide production in LV sections from sivelestat-treated hearts. Taken together, these results indicate that sivelestat infusion during postischemic low flow reduces infarct size and preserves vasoreactivity in association with decreased ROS formation and the preservation of nitric oxide.

## 1. Introduction

Reperfusion of the ischemic myocardium occurs in nearly 2 million people annually in the United States in patients experiencing cardiac arrest, myocardial infarction, or undergoing cardioplegic arrest during cardiac surgery [1]. Various levels of low flow are induced following such ischemic events, most notably in the early moments of reperfusion [2–4]. In the case of cardiac arrest, which affects over 300,000 people annually in the USA, cardiopulmonary resuscitation (CPR) is used by first responders to initiate reperfusion [5]. Currently this low flow reperfusion generated by CPR [6] results in a survival-to-hospital discharge rate for out-of-hospital cardiac arrest patients of <10% [7]. Periods of low coronary flow following myocardial ischemia may present early opportunities for pharmacologic treatment of ischemic myocardium prior to full flow reperfusion [8]. 

There is currently no approved drug on the US market for postischemic reduction of myocardial infarction [9]. The neutrophil elastase inhibitor sivelestat (ONO-5046) [10] has recently been shown to be cardioprotective in several animal studies and in at least one study in humans [11–13]. It has been recently demonstrated that sivelestat preserves LV function when infused after global ischemia in the Langendorf buffer-perfused heart, a model of the functioning heart, that is, bereft of neutrophils [14]. The neutrophil-independent cardioprotective mechanisms of sivelestat in the setting of ischemia-reperfusion (IR) are unknown.

Reactive oxygen species (ROS) appear to play an important role in reperfusion-induced injury [15], specifically superoxide, hydroxyl radical, and hydrogen peroxide [16]. The release of oxygen-derived free radicals concurrent with the reintroduction of oxygen contributes to the pathophysiology of IR injury [17, 18]. In addition to cellular and mitochondrial injury through oxygen radical-mediated damage, reactive oxygen species signal neutrophil infiltration and are implicated in endothelial dysfunction and smooth muscle injury [19–22]. Specifically, ischemia-reperfusion impairs endothelium-dependent vasorelaxation of coronary microvessels through production of ROS [23]. Excess hydrogen peroxide production during reperfusion damages vascular smooth muscle cells. Conversely reduction of ROS during reperfusion attenuates myocyte and endothelial cell injury following IR [24]. 

In this study, we tested the hypothesis that infusion of sivelestat during postischemic low flow would reduce infarct size and preserve endothelial and contractile function through an ROS-mediated mechanism. We used the isolated perfused rat heart model of IR and fluorescent microscopy in tissue slices, and electron paramagnetic resonance (EPR) imaging in cultured endothelial cells, to detect the formation of reactive oxygen species. Our results demonstrate a neutrophil-independent mechanism of sivelestat to reduce infarct size and preserve cardiac performance while reducing early ROS formation and preserving endothelial function.

## 2. Methods

### 2.1. Isolated Buffer-Perfused Rat Hearts

Male Sprague-Dawley rats (400–500 g) purchased from Harlan Laboratories (Indianapolis, IN, USA) were cared for in accordance with the National Institute of Health (NIH) guidelines and the approval of the Institutional Animal Care and Use Committee. Hearts were isolated and perfused with buffer as previously described [25]. Rats were anesthetized with intraperitoneal sodium pentobarbital (70 mg/kg) and heparin (1,000 U/kg). The trachea was cannulated with a 16-gauge angiocath attached to a rodent ventilator (Harvard Apparatus, South Natick, MA, USA). Animals were ventilated with room air at 70 respirations per min with a 2.5 mL stroke volume. A midsternal thoracotomy was performed to expose the heart and isolate the aorta. The aorta was cannulated in situ and hearts were excised. Retrograde perfusion of the coronary arteries was immediately initiated with warmed (37°C) modified Krebs-Henseleit buffer (1.25 mM CaCl_2_, 11 mM glucose, 112 mM NaCl, 25 mM NaHCO_3_, 5 mM KCl, 1.2 mM MgSO_4_, 1 mM K_2_PO_4_, and 0.2 mM octanoic acid, bubbled with 95% O_2_/5% CO_2_, pH 7.4) at a constant perfusion pressure of 75 mm Hg. A saline-filled latex balloon attached to a pressure transducer was inflated to 5–10 mm Hg in the left ventricle (LV) for measurements of LV contractile function. The heart was positioned inside a temperature-controlled glass chamber at 37°C.

### 2.2. Experimental Protocol

Global ischemia was induced by completely occluding perfusion flow to the heart. Hearts were randomly assigned to 3 ischemia groups (*n* = 8 per group): group 1: 25 min of global ischemia; group 2: 25 min of ischemia followed by 3 min of low flow with perfusion buffer at 4 mL per min; group 3: 25 min of ischemia followed by 3 min of low flow with sivelestat (100 *μ*g per mL) at 4 mL per min, administered through a side port directly above the aorta by a PHD 2000 Programmable pump (Harvard Apparatus, Holliston, MA, USA). All hearts were subsequently reperfused for 60 min at 75 mm Hg.

### 2.3. Coronary Flow and Left Ventricular Function

Coronary flow was continuously monitored by the use of an inline small animal flow meter (Model T206, Transonic Systems Inc., Ithaca, NY, USA) and recorded using an analog-to-digital converter box (Digi-Med ASA-400a, Micro-Med, Inc., Louisville, Kentucky, USA). LV pressure was continuously sampled at 30 Hz and digitally processed with a Digi-Med Heart Performance Analyzer (HPA-210a, Micro-Med, Inc.). Heart rate, dP/dt_max⁡_, LV systolic pressure, and LV end diastolic pressure were derived by computer algorithm. Developed pressure was calculated as the difference between systolic and end diastolic pressures. Rate pressure product (RPP) was calculated as the product of heart rate and developed pressure. Hearts that did not achieve an average RPP of at least 20,000 and an average dP/dt_max⁡_ of at least 2,500 in the preischemic baseline stabilization period were excluded from further experimentation. Metrics of LV functional recovery were calculated as percent recovery at the end of experimentation relative to baseline (preischemic) values.

### 2.4. Acetylcholine-Induced Change in Coronary Flow

Following 60 min of reperfusion, hearts were infused with 1.0 *μ*M acetylcholine chloride for one minute and then switched back to normal perfusion fluid. At this point the recovery in coronary flow was recorded as the maximum coronary flow value within one minute of reflow with normal perfusion fluid. Since perfusion pressure was maintained constant at 75 mm Hg, increases and decreases in coronary flow were a reflection of endothelial relaxation and constriction, respectively.

### 2.5. Infarct Size

Following IR, hearts were stained with triphenyltetrazolium chloride (TTC) for the measurement of infarct size, using the method of Ferrera et al. [26]. Under TTC staining, living tissue appears brick red and infarcted tissue appears pale pink or white. Hearts were frozen at −80°C, sliced in 2 mm sections, incubated in 1% TTC at 37°C for 10 min per side to allow mitochondrial uptake of TTC, and then fixed in formalin prior to photomicrography. Infarct size was measured using MetaVue imaging software (version 6.2r6, Universal Imaging Corp., Downingtown, PA, USA) and reported as a percent of the total left ventricular area.

### 2.6. Creatine Kinase Release

Coronary effluent was collected from all hearts before ischemia (i.e. in baseline) and at 10, 30, and 60 minutes of full reperfusion. The effluent was assayed for creatine kinase (CK) content using a standard spectrophotometric assay kit (Stanbio Laboratory, Boerne, TX). Values of CK activity are reported in U · L^−1^ [27].

### 2.7. Measurement of Superoxide in LV Sections by DHE Staining

In another block of experiments using duplicate groups, hearts were collected at the end of the 10 min of reperfusion for measurements of ROS production under fluorescent microscopy, as previously reported [28]. After reperfusion, hearts were embedded in optimal cutting temperature gel, sliced to 5 *μ*m thick in a cryotome, and placed on glass slides. After application of 10 *μ*M dihydroethidium (DHE), tissue sections were incubated in a light-impermeable chamber at 37°C for 30 min. In the presence of superoxide DHE is converted to the red fluorescent hydroxyethidium molecule [29]. Slides were costained with 4′,6-diamidino-2-phenylindole (DAPI) and photographed using a Nikon Eclipse TE 2000-U microscope (Tokyo, Japan) equipped with an X-Cite 120 Fluorescence Illumination System (Lumen Dynamics Group Inc., Mississauga, Ontario). Photographs of hydroxyethidium fluorescence were taken under a rhodamine filter (green excitation 550 nm, red emission 573 nm). Fluorescent intensity, which positively correlates with superoxide generation in tissue, was quantified using MetaMorph image analysis software (Molecular Devices, Sunnyvale, CA, USA).

### 2.8. Effect of Nitric Oxide Synthase (NOS) Inhibitor in Isolated Hearts

Additional hearts were isolated to determine if sivelestat exerts cardioprotection in the presence of NOS inhibition. Isolated hearts (*n* = 4 per group) were subjected to 25 min of ischemia and 3 min of low flow at 4 mL per min followed by full reperfusion for 120 minutes. During 3 min of low flow, hearts were directly infused with either (a) perfusion buffer; (b) sivelestat (100 *μ*g per mL); (c) the nonspecific NOS blocker N-nitro-L-arginine methyl ester (L-NAME, 100 *μ*M); or (d) L-NAME + sivelestat (*N* = 4 per group). LV function and infarct size were recorded as detailed above. 

### 2.9. Effect of Hypoxia-Reoxygenation in Bovine Aortic Endothelial Cells

To determine the influence of sivelestat on ROS production in endothelial cells, cultured bovine aortic endothelial cells (BAECs) were subjected to hypoxia-reoxygenation in the presence of the spin trap 5,5-dimethyl-1-pyrroline-N-oxide (DMPO) [17]. BAECs were cultured in low-glucose DMEM supplemented with 10% FBS, penicillin/streptomycin, and 0.1% of Endothelial Cell Growth Supplement (Millipore, Billerica, MA, USA). Cells were cultured in a humidified environment of 5% CO_2_/21% O_2_. When cells achieved 80–85% confluence, they were washed, trypsinized, pelleted, and suspended in low-glucose DMEM without phenol red to achieve a cell count of 5 × 10^6^ cells per mL. To achieve hypoxia, cells were placed in a heated chamber at 37.0°C and flushed with 100% nitrogen for 45 min. Immediately following the rapid addition of DMPO (50 mM final concentration), endothelial cells were flushed with oxygen (95% O_2_/5% CO_2_, pH 7.4) for one minute at 37.0°C in the presence of (a) no drug, (b) sivelestat (100 *μ*g per mL final concentration), (c) the xanthine oxidase inhibitor oxypurinol (500 *μ*M), or (d) superoxide dismutase (SOD, 1 kU per mL from bovine erythrocytes (Sigma-Aldrich Co., St. Louis, MO, USA). Cells were then transferred to a quartz flatcell inside an EPR-300 X-band (9.7 GHz) spectrometer. Second peak amplitude of the DMPO-OH spectrum was quantified and taken as an indication of the magnitude of superoxide production.

### 2.10. Data Analysis

Data was expressed as mean ± SEM. Statistical significance between groups was calculated by one-way ANOVA followed by the Tukey range test for multiple comparisons. A *P* value of <0.05 was considered statistically significant.

## 3. Results

### 3.1. Sivelestat Preserved LV Function

**Figure 1:**
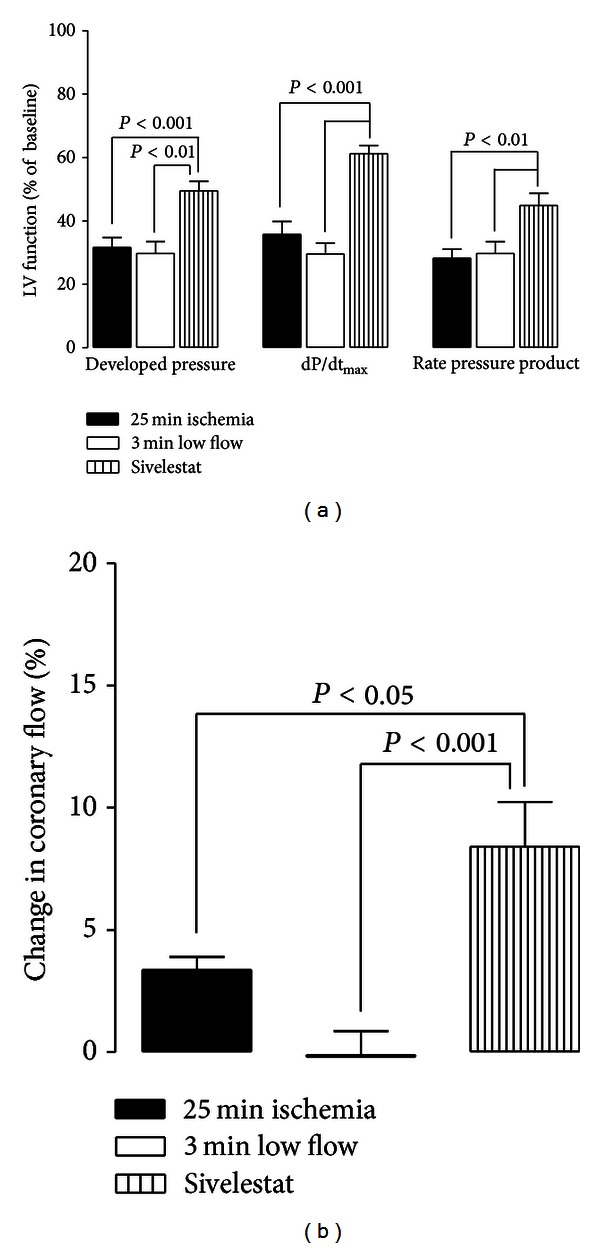
Sivelestat preserves LV and coronary vascular function in ischemia-reperfusion. (a) Effect of sivelestat on LV function and infarct size. Sivelestat significantly improved recovery of developed pressure, dP/dt_max⁡_, and rate pressure product (RPP) as compared to hearts that did not receive low flow (25 min isc) (*n* = 8 per group). (b) Rebound in coronary flow. Sivelestat significantly preserved vasoreactivity at the end of reperfusion as shown through the rebound in coronary flow after one minute of acetylcholine (1 *μ*M) infusion (*n* = 4/group).

Administration of sivelestat during 3 min of low flow following ischemia improved the recovery of developed pressure, dP/dt_max⁡_, and rate pressure product (RPP) at 60 min of reperfusion as compared to hearts receiving 25 min of ischemia or low flow without sivelestat (Figure 1(a)).

### 3.2. Sivelestat Administration Preserved Vasoreactivity

The vasoconstrictor acetylcholine was infused for one min at the end of 60 min of reperfusion. Following this, normal buffer was reperfused, and the rebound in coronary flow was taken as a metric of vasoreactivity. Administration of sivelestat significantly improved recovery of coronary flow (CF) following acetylcholine infusion (8.4 ± 1.8%) as compared to hearts receiving 25 min of ischemia (3.4 ± 0.50%) or low flow without sivelestat (−0.20 ± 1.0%) (Figure 1(b)).

### 3.3. Sivelestat Decreased Myocardial Infarct Size

**Figure 2:**
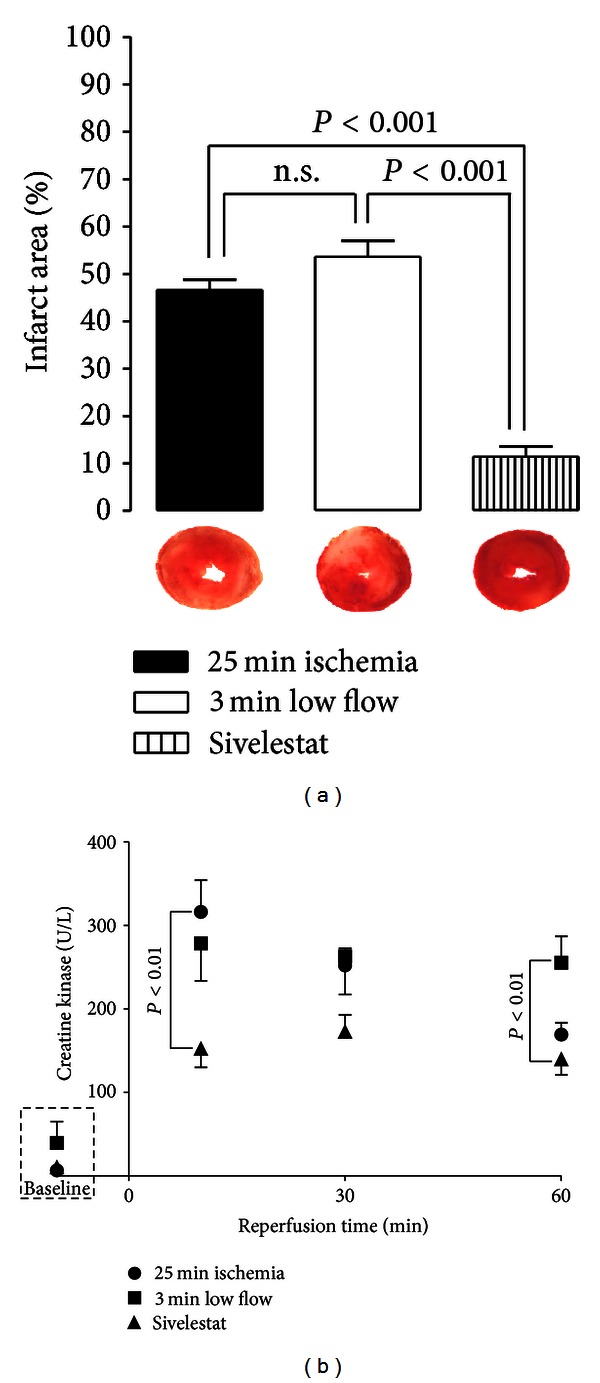
Sivelestat preserves LV tissue in ischemia-reperfusion. (a) Percent infarct area with representative images of left ventricle slices. Sivelestat significantly reduced infarct area as compared to hearts given 25 min ischemia or ischemia and low flow with vehicle only, as observed through TTC staining. Whiter areas indicate regions of tissue infarction, and pink and red areas indicate functional tissue (*n* = 4 per group). n.s.: no significance. (b) Extrusion of intracellular creatine kinase into coronary effluent. Creatine kinase (an intracellular enzyme) release is indicative of cell membrane rupture. Creatine kinase release (U/L) was significantly reduced by treatment with sivelestat at 10 min of full reperfusion, as compared to hearts that did not receive low flow, and at 60 min of reperfusion, as compared to vehicle-treated low flow hearts (*n* = 8/group).

At the end of 60 min of reperfusion, hearts infused with sivelestat during 3 min of postischemic low flow had significantly lower infarct area (11 ± 2.2%) than hearts given normal perfusion buffer during low flow (54 ± 3.4%) and hearts receiving 25 min of ischemia with no low flow (47 ± 2.1%). Representative images are shown in Figure 2(a).

### 3.4. Sivelestat Decreased Myocardial Creatine Kinase Levels

Creatine kinase (CK) release was measured in coronary effluent collected during baseline and at 10, 30, and 60 min of reperfusion. CK was significantly reduced at 10 min of reperfusion in hearts treated with sivelestat during 3 min of postischemic low flow as compared to hearts that received 25 min of ischemia with no low flow (Figure 2(b)).

### 3.5. Inhibition of eNOS with L-NAME Reversed Cardioprotection from Sivelestat

**Figure 3:**
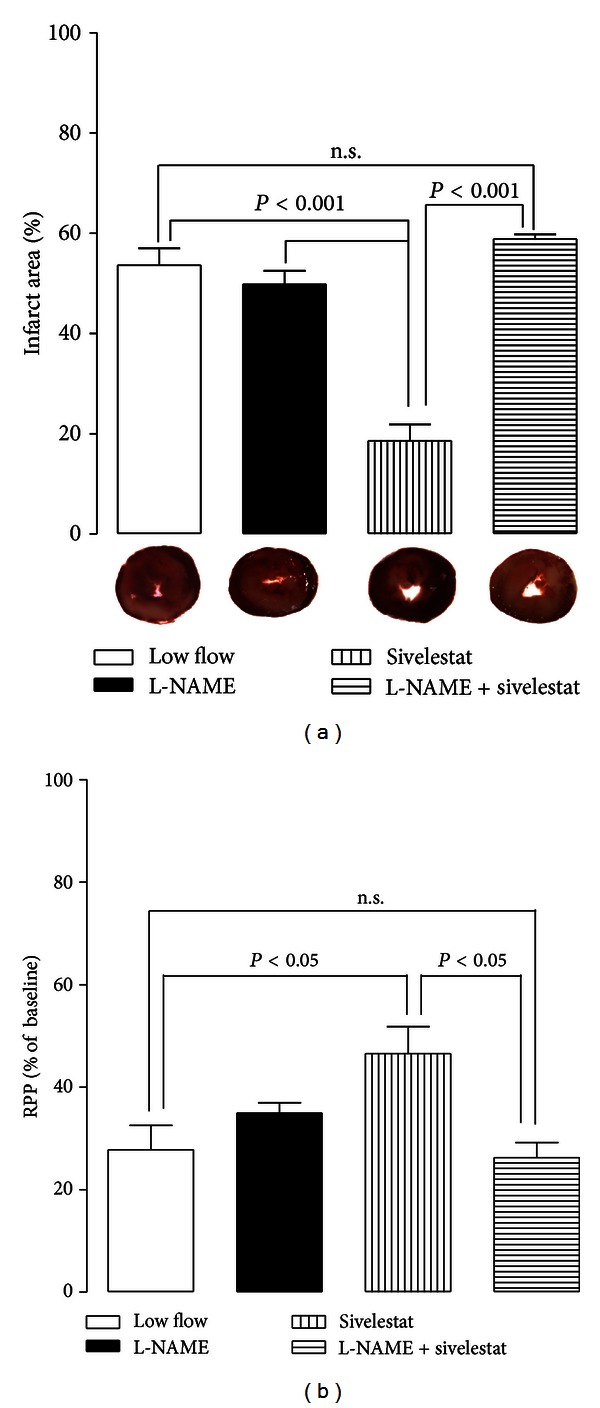
L-NAME reverses the cardioprotective effects of sivelestat in ischemia-reperfusion. (a) Infarct size and (b) recovery of rate pressure product. During low flow, hearts were treated with L-NAME, sivelestat, or both. Though sivelestat preserves RPP and reduces infarct following IR, coinfusion of L-NAME and sivelestat offered no preservation of RPP or infarct reduction as compared to hearts that received vehicle during low flow (*n* = 4/group). n.s.: nonsignificant.

After 2 hours of full reperfusion the percent infarct size was significantly less in hearts treated with sivelestat compared to L-NAME-treated hearts (sivelestat I.S. = 19 ± 3.2% versus L-NAME I.S. = 50 ± 2.7%). This sivelestat-mediated reduction in infarct size was lost in the L-NAME + sivelestat-treated hearts (I.S. = 59 ± 0.97%) (Figure 3(a)). In these same hearts, the percent recovery of rate pressure product (RPP) was significantly greater in sivelestat-treated hearts compared to vehicle-treated hearts (sivelestat RPP = 47 ± 5.3% versus vehicle RPP = 28 ± 4.7%) and to L-NAME + sivelestat-treated hearts (RPP = 26 ± 2.9%) (Figure 3(b)). 

### 3.6. Sivelestat Decreased Superoxide Levels in LV Tissue Sections

**Figure 4:**
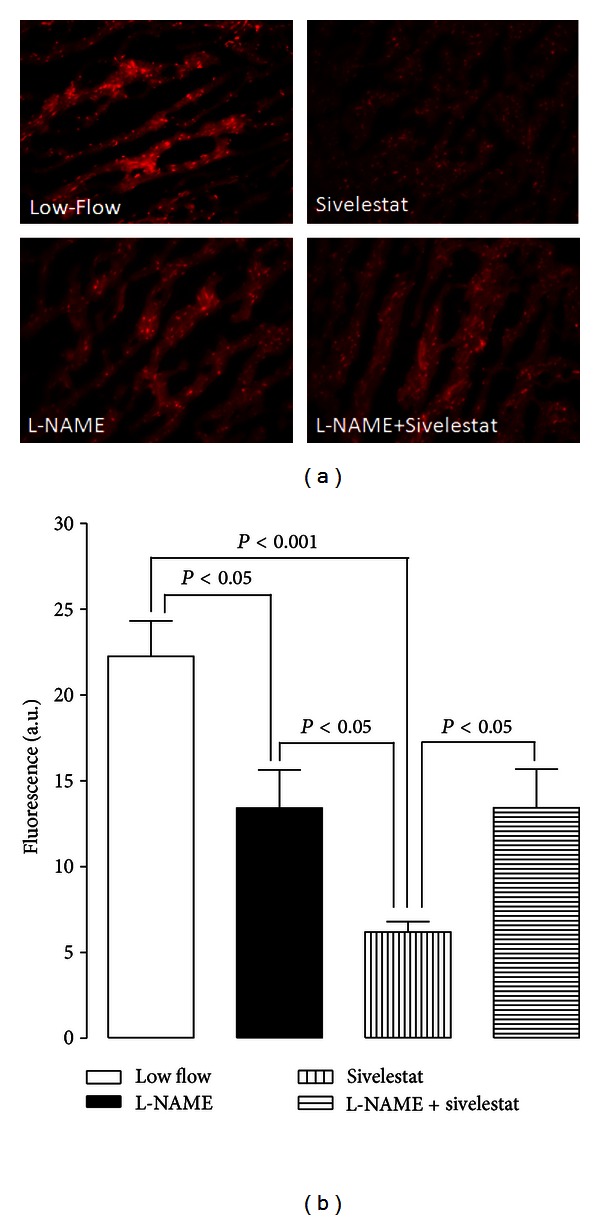
Measurement of ROS in the heart by DHE fluorescence. (a) Representative confocal fluorescence images of cryopreserved LV sections from different groups stained with DHE. (b) Quantification of superoxide generation is indicated by DHE fluorescence, sivelestat decreased tissue ROS during reperfusion, as compared to hearts infused with L-NAME. This decrease was reversed when L-NAME was coinfused with sivelestat (*n* = 3–5 per group). n.s.: nonsignificant.

The blockade of eNOS by L-NAME reversed the effect of sivelestat by increasing superoxide fluorescence (Figure 4(a)). DHE staining showed that superoxide generation was significantly decreased in the sivelestat group (Figure 4(b)). In contrast an increase in superoxide fluorescence was seen in the low flow, L-NAME, and L-NAME + sivelestat treated groups. This data demonstrates the involvement of nitric-oxide-mediated cardioprotection by sivelestat.

### 3.7. Sivelestat Decreased ROS Formation in BAECs

**Figure 5:**
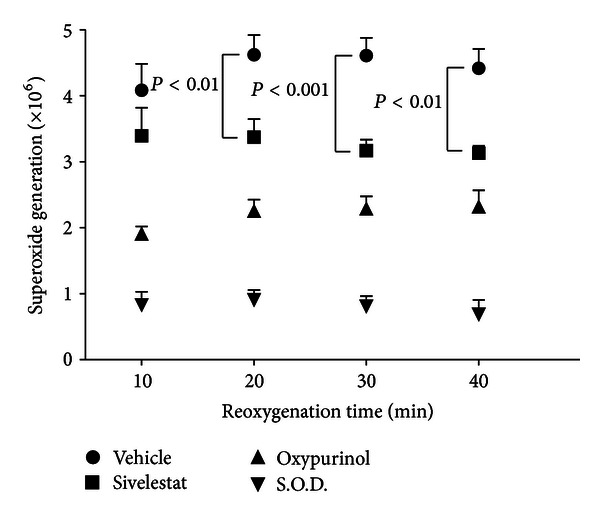
EPR spin-trapping of superoxide with DMPO in hypoxic-reoxygenated bovine aortic endothelial cells. First integral of DMPO-OH, amplitude of the 2nd spectral peak. DMPO-OH is the stable EPR-active adduct of trapped superoxide. All drugs were applied prior to hypoxia. Sivelestat (190 *μ*M) decreased the formation of superoxide (DMPO-OH) in bovine aortic endothelial cells as indicated by spin-trapping with DMPO. Oxypurinol (500 *μ*M) was more effective than sivelestat, and S.O.D. (5 kU/mL) was most effective at decreasing DMPO-OH (*n* = 5 per group).

To measure the production of superoxide by hypoxic-reoxygenated bovine aortic endothelial cells (BAECs), DMPO was added at the moment of reoxygenation as a spin trap for superoxide. The EPR-active molecule DMPO-OH is the molecular product of superoxide trapped by DMPO. Sivelestat reduced oxygen radical formation in hypoxic-reoxygenated BAECs exposed to 45 min of hypoxia followed by reoxygenation. As indicated by the 2nd peak height in the 1st integral of the DMPO-OH EPR spectrum, the spin adduct DMPO-OH was significantly reduced in cells treated with 100 *μ*g/mL sivelestat, as compared to untreated endothelial cells, after 20 min, 30 min, and 40 min of reoxygenation. Oxypurinol significantly reduced DMPO-OH formation at every time point as compared to vehicle, but not as effectively as S.O.D. (Figure 5).

## 4. Discussion

 In this study, the combination of postischemic low flow with a novel pharmaceutical agent, sivelestat, for 3 minutes at the onset of reperfusion successfully reduced infarct size, improved contractile function, preserved endothelium-dependent vasodilatation, and reduced creatine kinase release, an indicator of tissue destruction at the cellular level [30]. Sivelestat reduced superoxide production in both the ischemia-reperfused explanted LV tissue and the hypoxia-reoxygenated cultured endothelial cells. However, the functional cardioprotection and reduction in tissue superoxide generation effected by sivelestat was lost with NOS inhibition. Our study is the first to demonstrate the infarct-reducing property of sivelestat in the ischemia-reperfused heart.

Preservation of LV contractile function is in agreement with Kambe et al., who showed a 35% recovery of LV developed pressure versus controls (16%) when sivelestat was infused continuously during the first 10 min of full reperfusion in isolated rat hearts [14]. Also in agreement with Kambe et al., sivelestat significantly preserved endothelium-dependent vasorelaxation following IR. That study showed a 15% increase over controls in the rebound in coronary flow in response to acetylcholine infusion at the end of reperfusion in hearts treated with sivelestat. 

The infarct-sparing effects of sivelestat were reversed with simultaneous NOS enzyme inhibition, suggesting that nitric oxide bioavailability is essential during a brief 3-minute period of postischemic low flow. It is likely that sivelestat protects endothelium through dependence on functional NOS enzymes since myocardial protection was abolished by NOS blockade in our study. The idea that preservation of NO bioavailability may lead to myocardial salvage from IR has been widely accepted [31]. Others have shown that sivelestat requires functional NOS enzymes for exertion of protective effects on tissue. Okajima et al. concluded that sivelestat reduced IR-induced liver injury, an effect that was completely inhibited by pretreatment with L-NAME and relied on endothelium-dependent production of NO [32]. Takayama and Uchida showed that sivelestat inhibited substance P-induced contraction of tracheal ring preparations and that this effect was significantly attenuated by either removal of epithelium or blockade of NOS by L-NAME. However, they concluded that sivelestat exerted vasodilatory effects on guinea pig airways by both NO- and epithelium-dependent and NO- and epithelium independent mechanisms [33].

The retention of endothelium-dependent vasoreactivity with sivelestat may be explained by preservation of sensitivity to nitric oxide in the smooth muscle. However, other studies assert that sivelestat mediates vasorelaxation independent of endothelium. Maeda et al. suggested that this mechanism lies in the vasculature, by demonstrating that sivelestat selectively inhibits calcium sensitization to a receptor agonist in porcine vascular smooth muscle strips with or without endothelium, without affecting calcium-induced contraction [34]. Amemori et al. reiterated this work showing that sivelestat induces endothelium-independent vasorelaxation in precontracted human gastric arteries [35]. It should be noted that sivelestat also protects the heart in situations where neutrophils are present. Akiyama et al. showed attenuation of myocardial stunning in swine with postischemic infusion of sivelestat [11]. Toyama et al. reported an association of sivelestat infusion with improved fractional area of change in the left ventricle of pediatric patients who underwent cardiovascular surgery with cardiopulmonary bypass, demonstrating that sivelestat is protective in at least one setting of ischemia-reperfusion in humans [12]. However, our results confirm that sivelestat preserves vasodilatation in the coronary endothelium of hearts subjected to IR in an environment without neutrophils.

**Figure 6:**
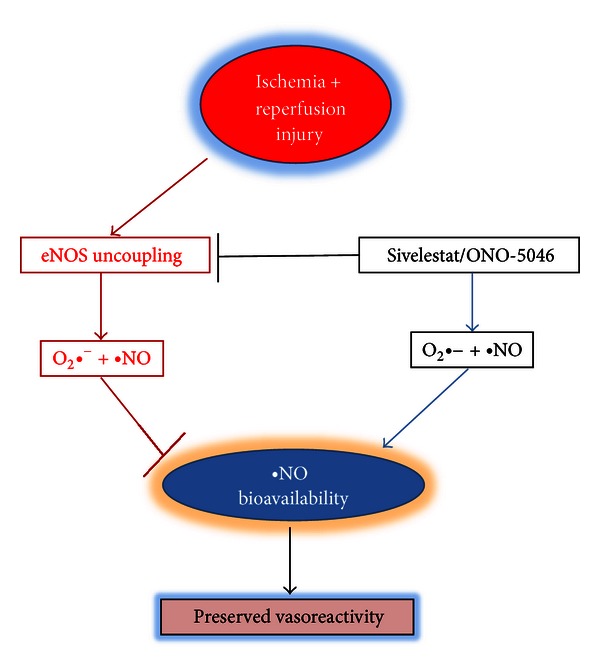
Proposed mechanism of sivelestat cardioprotection. During reperfusion, eNOS enzymes are uncoupled from nitric oxide production and produce superoxide in excess. Sivelestat may prevent eNOS uncoupling, thereby reducing superoxide overproduction and preserving nitric oxide production. Nitric oxide bioavailability preserves vascular function in cardiac ischemia-reperfusion.

The role of eNOS in sivelestat-induced cardioprotection must be further explored. In order to produce nitric oxide from L-arginine, eNOS enzymes require tetrahydrobiopterin (BH_4_) to couple L-arginine oxidation to NADPH consumption and prevent dissociation of the ferrous-dioxygen complex [36]. When BH_4_ is depleted, as in myocardial ischemia, the association of the ferrous-dioxygen complex can decrease. Once perfusion is resumed, eNOS is not able to couple L-arginine oxidation to NADPH consumption and becomes a true NADPH oxidase, producing superoxide instead of nitric oxide [37]. It is reasonable to hypothesize that sivelestat preserves the association of the ferrous-di-oxygen complex thus preserving nitric oxide bioavailability. In support of this hypothesis we noted an increase in vascular reactivity to acetylcholine and a reduction of superoxide production in aortic endothelial cells (see Figure 6). However, additional studies are needed to elucidate the exact mechanism(s) of protection exerted by sivelestat. For instance, direct measurements of NO production by coronary endothelium are needed to confirm that sivelestat enhances bioavailability of NO.

In summary, our results demonstrate that sivelestat protects the heart against IR injury by scavenging ROS and thereby decreasing the oxidative damage at reperfusion leading to improved LV function and decreased infarct size. The cardioprotective effect of sivelestat may be attributed to an NOS-mediated mechanism. Sivelestat is effective at salvaging tissue when applied in very brief postischemic low flow conditions. This low flow reperfusion differs from the classic mechanical postconditioning, which consists of stuttered start-stop episodes following ischemia [38]. 

## 5. Conclusions

Overall, these results demonstrate that the period of postischemic low flow can be exploited for significant cardioprotection by pharmaceutical intervention. Due to its availability, promising results in animal studies, and current use in humans, sivelestat is a promising translational pharmaceutical for the investigation of myocardial infarction treatment.

## Abbreviations

ROS: Reactive oxygen species
IR: Ischemia-reperfusion
LV: Left ventricular
BAEC: Bovine aortic endothelial cell
CK: Creatine kinase
RPP: Rate pressure product
TTC: Triphenyltetrazolium chloride
DAPI: 4′,6-Diamidino-2-phenylindole
DHE: Dihydroethidium
DMPO: 5,5-Dimethyl-1-pyrroline N-oxide
ERP: Electron paramagnetic resonance
ONOO^−^: Peroxynitrite
NOS: Nitric oxide synthase
L-NAME: L-Nitro-arginine methyl ester
O.D.: Optical density.
